# Carney-Complex: Multiple resections of recurrent cardiac myxoma

**DOI:** 10.1186/1749-8090-6-12

**Published:** 2011-02-03

**Authors:** Christian Bireta, Aron F Popov, Hanna Schotola, Brian Trethowan, Martin Friedrich, Mohamed El-Mehsen, Friedrich A Schoendube, Theodor Tirilomis

**Affiliations:** 1 Department of Thoracic and Cardiovascular Surgery, University of Göttingen, Germany; 2 Department of Cardiothoracic Transplantation & Mechanical support, Royal Brompton & Harefield Hospitals, London, UK; 3 Department of Anaesthesiology, Emergency and Intensive Care Medicine, University of Göttingen, Germany; 4 Department of Critical care & Anaesthesia, Royal Brompton & Harefield Hospitals, London, UK

## Abstract

We report a case of a female patient who was operated at the third relapse of an atrial myxoma caused by Carney complex. The difficult operation was performed without any complications despite extensive adhesions caused by the previous operations. The further inpatient course went without complications and the patient was discharged to the consecutive treatment on the 9th postoperative day. The echocardiographic finding postoperative showed no abnormalities.

## Case report

In 1998 a 45 year old female patient presented with complete left sided hemiparesis after a cerebral embolism. While focusing on the reason for this clinical presentation a large mass lesion was found in the atrium via echocardiography whereby suspicion of atrial myxoma was raised. The patient was referred to our department and operative resection of the large myxoma was performed which was predominant prolapsed into the left ventricle at the distal roof of the left atrium above the mitral valve. Both intraoperative and inpatient course progressed without complications and the patient was rapidly discharge for early neurological rehabilitation.

Three years later, a left atrial tumor was seen in a routine cardiological echocardiography follow up and the suspicion of a relapse of the atrial myxoma was raised. A resection of the two-hazelnut-sized myxoma at the posterior wall of the left atrium was performed again. The operation and inpatient course again proceeded without complications and the patient was rapidly discharged again.

After a period of four years an echopenic structure with freely moving component parts was found at the left side during a transesophageal echocardiography and the suspicion of a second relapse was raised. Making additionally there were found sutures of the previous operations at the septum. But making a clear differentiation between a thrombus at the previous suture line and a myxoma relapse was not possible; therefore a three months oral anticoagulation with phenprocoumon was started.

With a positive family history for myxomas (the patients's brother and grandfather) and multiple lentigines an evaluation of the presence of Carney complex, an autosomal dominant disorder, was conducted. After genetic analysis the diagnosis of Carney complex type I with a deletion mutation in the PRKAR1A gene was detected. Carney complex type I is characterized by recurrent atrial myxomas, skin, conjunctiva and lips lentigines, subclinical hypercortisolism and nodular thyroid changes.

**Figure 1:**
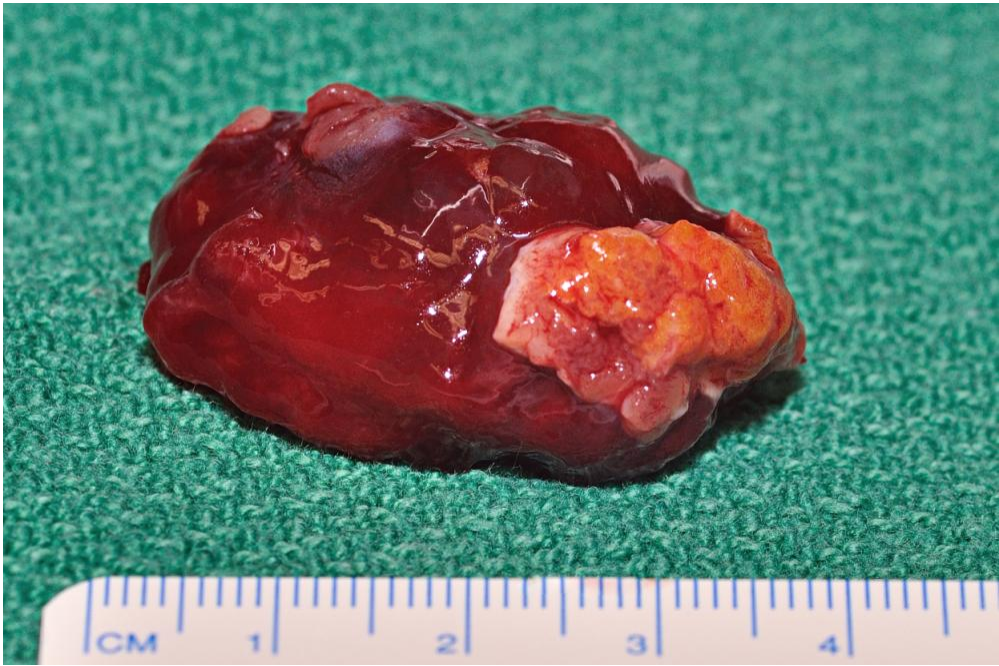
**Intraoperative finding of the resected myxoma**.

After three months of effective oral anticoagulation the structure at the atrial septum was not increased in size. A conservative approach was agreed while maintaining oral anticoagulation and regular echocardiographic follow up. Three years later an echocardiographic study revealed a new tumor, however this time the location was the right atrium. The left sided structure was unchanged. An operative resection was carried out again via re-sternotomy with an oscillating saw after preoperative evaluation (computed tomography (CT) scan of the thorax, transthoracic doppler echocardiogram, doppler examination of the femoral vessels, and lower limb arteries). The cardiopulmonary bypass with systemic 32°C mild hypothermia was established via ascending aortic and bicaval cannulation. The right atrial tumor (approximately 3 cm in diameter) was located at the confluence of inferior vena cava and the lateral atrial wall. An atrial myxoma was confirmed histologically with a tumour-free resection margin. The postoperative course was again without complication. During the last echocardiographic follow up a progressive left atrial structure was seen and the decision was made to resect the fourth atrial myxoma (Figure 1). Again the postoperative course went without complications.

## Discussion

As with every other organ the heart can be affected by tumor and its relapses. Primary tumors of the heart are rare. The incidence varies between 0.0017 and 0.19% in unselected autopsy studies. Three quarters of primary heart tumors are benign and half of them are atrial myxomas. Other benign tumors are lipoma, papillary fibroelastoma, rhabdomyoma and fibroma [1,2].

Myxomas are mesenchymal tumors, which can occur at any age, however, they mainly exist between the 30th and 60th year of life. This disease is approximately two to three times more prevalent in women than in men [1-3]. About 75% of the myxomas are located in the left atrium, 20% in the right atrium and 5% in one of the ventricles. Multiple tumors in different ventricles have been described in a few patients [1,3]. Myxomas are typically sporadic and isolated, only in around five percent of all cases this disease occurs as a familial disease [1]. This group of patients are younger, frequently have multifocal tumors, but, there is no gender preference. Because every fifth patient suffers from additional neoplasms this disease pattern is also called 'complex myxoma' or Carney complex [1,4,5]. Most of the myxomas are pedunculated (seldom broad based) and are often located in the area of fossa ovalis, but they can also occur anywhere in the atrial wall, the vena cava or rarely at the heart valves [1-3]. The tumor's mobility depends mostly on the length of the tumor stem. The clinical picture is determined by localization, dimension, condition and tumor mobility [1,2]. Characteristically, the period until the diagnosis is made is highly variable, because the time free of symptoms can be quite long. Usually a local complication leads to symptoms and this requires further diagnostic tests. Most common complications are embolism (about 30-40%, both peripheral and central, whereas central embolisms are more frequent), intra cardiac obstruction and obstruction of the ventricular outflow tract (seen in left or right heart failure with symptoms of dyspnoea, syncope, supra ventricular cardiac arrhythmia). Another common complication is the so called 'myxoma disease' with fever, arthralgies, polymyositis, weight loss and hypergammaglobulinaemia [1-3,6].

The clinical examination, chest X-ray and ECG are inefficient and non-specific. Often the diagnosis is an incidental finding detected by echocardiography measurements in the context of peripheral tumor emboli diagnosis. However, transthoracic or transesophageal echocardiography can show exactly the tumor's position and dimension, alternatives are cardiac CT and cardiac MRI. Differential diagnosis to consider is always a thrombus giving the same clinical picture. The preferred treatment is always surgical removal indicating a curative approach. Both short- and long-term results are excellent [1-3].

An operation is always indicated because the outcome and clinical course without intervention cannot be predicted. Due to the high rate for embolisms and the danger of the sudden cardiac death the operation should be performed as soon as possible following diagnosis [1-3]. The risk of a myxoma relapse is well documented in the literature. Reasons for relapses are: inadequate resections, intraoperative implantation of parts of the tumor and multi-located tumor origin. The relapse risk for sporadically occurring myxomas varies between 1 and 3% and it is increased significantly for patients with familial aggregation or Carney complex [1,3,7]. The Carney complex (CNC) is an autosomal dominant inheritable disease characterized by myxomas, schwannomas, germ cell tumors, abnormal skin pigmentation and endocrine hyperactivity. The average age at the time of diagnosis is around the age of 20 [4,8-10]. There is no gender-related difference and the Carney complex can occur in all races. In 50% of the patients there is a mutation in the PRKAR1A gene on chromosome 17 (type I or CNC1). This gene codes for the regulatory subunit type 1A of protein kinase A, that plays an important role in different endocrine signaling cascades and also as a tumor suppressor gene. A mutation on chromosome 2 probably codes for a, so far, not characterized variation of the Carney complex (type II of CNC2) [8,10]. The life expectancy for patients with Carney complex is reduced. Most of the patients die from complications of cardiac myxomas, metastasizing or intra cranial psammomatous melanotic schwannomas, thyroid carcinomas and metastasising pancreatic or germ cell tumors [8]. Hence, close monitoring is necessary for patients with Carney complex and their blood relations. With an additional genetic analysis the diagnosis of Carney complex was made for our patient.

As with sporadically occurring myxomas a resection of the cardiac tumor and its relapses should be performed in patients with Carney complex. Serious surgical problems are especially peri- and epicardiac adhesions from previous operations which increase after every relapse resection. This can lead to a situation where the surgical risk is higher than the risk of death due to complications of the myxoma. Therefore a careful consideration of the complication-benefit analysis should be made especially for patients with Carney complex. In individual cases a non-operative approach has been described [7]. In the event of the second relapse we also recommended a non-operative approach combined with continued anticoagulation as prophylaxis against apposition thrombi and careful clinical examination. Our reasoning consisted of the two previously performed operations were the dimension, localization and morphology of the tumor relapse. However, due to the increase in the dimension of the lesion and appearance of cardiac symptoms we decided to resect the myxoma relapse for a fourth time.

Normally echocardiographic follow up should be performed each year to detect myxoma relapses early. Patients with known Carney complex should have this examination every six month if they have already had a surgical resection [8,9]. After the fourth operation due to the third myxoma relapse we considered platelet aggregation inhibition for our patient as a treatment to prevent complications associated with emboli as sufficient.

## Conclusion

The Carney complex is an infrequent congenital disease where the relapse rate of cardiac myxomas is manifestly increased. With close follow up with echocardiography relapses can be detected early and adequately treated. Concerning postoperative anticoagulation we considered it appropriate that our patient received a platelet aggregation inhibitor in light of present of inconspicuous echocardiographic findings.

## Competing interests

The authors declare that they have no competing interests.

## Authors' contributions

CB and AP performed data, and wrote the paper. ME, MF, and TT are members of surgical teams. HS was the anaesthetist involved in theatre and in intensive care unit. BT an FS added import comments to the paper. TT co-wrote the manuscript. All authors have read and approved the final manuscript

## Consent

Written informed consent was obtained from the patient for publication of this case report and any accompanying images. A copy of the written consent is available for review by the Editor-in-Chief of this journal.
